# Brief Notice of a Case of Moral Insanity, Unaccompanied by Any Obvious Symptoms of Intellectual Aberration
*Read before the Association of the College.


**Published:** 1853-04-01

**Authors:** James F. Duncan

**Affiliations:** FELLOW AND CENSOR OF THE IRISH COLLEGE OF PHYSICIANS, PHYSICIAN TO SIR P. DUN'S HOSPITAL, DUBLIN


### BRIEF NOTICE OF A CASE OF MORAL INSANITY, UNACCOMPANIED BY ANY OBVIOUS SYMPTOMS OF INTELLECTUAL ABERRATION.*

BY JAMES F. DUNCAN, A.M., M.D, FELLOW AND CENSOR OF THE IRISH COLLEGE OF PHYSICIANS, PHYSICIAN TO SIR P. DUN'S HOSPITAL, DUBLIN.

Most of the cases of moral insanity which are met with in practice occur in connexion with monomania, where the perversion of the moral feelings occupies but a subordinate place in the morbid phenomena, and where the character of the disease is sufficiently established by the obvious lesion of the intellectual faculties.

Whether such a thing ever occurs as a case of real insanity, unaccompanied by a morbid condition of the reasoning powers, properly so called, is doubted by many practitioners; and it is certainly no part of my present intention to maintain the affirmative of this question; but that cases do occasionally occur in which such disorder, if it exist at all, is not obvious enough to attract attention, is, in my opinion, sufficiently capable of proof. It is with the view of illustrating and elucidating this interesting subject that I have thought it might be worth while to lay before the Association a brief notice of a case which has lately fallen under my observation.

Mr. S. G., a gentleman about forty years of age, and a member of the Society of Friends, was the son of a respectable merchant in this city. As far as I cn conjecture, he was tenderly brought up; and though not an only child, he was greatly indulged by both his parents, but especially by his mother. This circumstance I believe had much to do with the peculiar turn which his history afterwards took. It is not difficult to understand how over-indulgence in early life must lead to much misery afterwards, by fost ering feelings which ougt to be kept in check, and enfeebling affections

* Read before the Association of the College.

which require to be encouraged. Our passions, like every other part of our mental constitution, become powerful by exercise, and are atrophied by disuse. The constant indulgence of every childish whim necessarily produces selfishness in him who is exposed to it, and impresses him with the consciousness of *power* over others, so as to extract at will whatever he sets his heart on, from his weak but affectionate parent. It is easy to see that whenever such results as these are produced, the individual is placed in a false position, in reference to society, and the foundation is laid for a train of feelings which may easily degenerate into insanity.

At the age of seventeen or eighteen, Mr. G. had a severe attack of scarlatina, after which he had a protracted convalescence, marked, as it was supposed, by the loss of the use of his lower extremities. But this, it appears, was only a make-believe, inasmuch as he told Mr. Tallis, the apothecary to Sir P. Dun's Hospital, that he could have walked well enough at the time, had he been disposed to do so. I infer, therefore, that it was a desire to enjoy a longer vacation from his lessons—natural enough in boys of his age—that induced him to carry on this deception.

On his father's death, he left the residence of his mother, who occupied a comfortable house in one of the most airy streets on the north side of the city, and went to live *en garçon* at his place of business in Pill-lane. The reason he assigned for this change was that he could not get his health in his mother's house, because none of the food he took there was properly digested. I believe that this was another instance of deception on his part, and that the real reason was a wish to be independent of parental control—common to most young men of his age,—and to cast off the strict habits of the religious denomination with which he was connected. In his new abode he told me he lived freely, saw a great deal of company, and committed other excesses.

For the last twelve or fifteen years he left the entire management of his business to a brother, who was his partner, and, under the plea of illness, retired to the neighbourhood of Killiney, where he lived very much by himself. The disease under which he was said to labour, was an affection of the spine, for which the most eminent members of the faculty were consulted, who prescribed a variety of treatment. Among other things, by direction of Sir P. Crampton, he made two voyages to Malaga, the first of which he thought was productive of advantage, but the second was not. During the last two years he had lost the entire use of his limbs, and in consequence was confined altogether to bed. In the early part of last year he had an attack of neuralgia, the pain of which was so severe as to oblige him to have recourse to large doses of laudanum for its alleviation. He cried out incessantly, and in a loud tone of voice, from the agony which this affection caused him. When somewhat relieved from this state, he was brought into town, to his mother's house, where for a time he was waited on with untiring diligence by his two sisters and a nurse. Day and night he was unrelenting in his exactions upon their attentions. He was never satisfied unless he had two of them present with him at a time, and if ever the second would attempt to leave him he was sure to call her back to perform some little office about his person. He manifested a total disregard to the feelings or comforts of others, not excepting his mother, who was old and infirm, and who occupied an adjoining room. On being asked at times to try and restrain his feelings, out of regard to her comforts, he has replied, "that is the very reason why I will cry out the more." All this time, no evidence of intellectual derangement could be detected: his memory was perfect, his mind clear, his language accurate. At last the paroxysms of shouting instead of being occasional, became constant, so much so, that the neighbourhood was disturbed as well as his own family. It became necessary, therefore, to have him removed to a distance; and under the idea that he was not a fit subject for a lunatic asylum, admission was sought for him into the pay wards of Sir P. Dun's Hospital,

A circumstance occurred on the occasion of his removal which throws considerable light on the nature of the case. As his two sisters accompanied him in a covered car, they told him, in answer to his inquiry as to where they were taking him, that as I had left home that morning when they called, it was necessary to take him to the place where I had gone to. Immediately on turning into Westland-row, he cried out, "I know where you are taking me to! you are bringing me to Sir P. Dun's Hospital. Take me home, and I promise you I will never shout out again as long as I live."

On visiting him at the hospital on the 29th of November, 1832, the day of his admission, I found him in appearance older by at least ten years than the age assigned to him; he was sitting up in bed, of a sallow complexion, somewhat emaciated, but otherwise not looking out of health; the pupils of his eyes—but particularly the right—somewhat dilated, though exposed to the full light of the window, and the levator palpebræ superioris of the left eyelid slightly paralyzed. The tongue was clean, and protruded perfectly straight. There was no loss of motor power or feeling in either arm. He complained of no pain in any particular spot; and though he said he had paroxysms coming on at times, he was unable to fix upon the locality. Both lower extremities were greatly emaciated, and the muscles flabby to a degree. No evidence of tenderness in any part of the spine could be detected, and there was complete power over both sphincters. The urine was healthy, and the bowels habitually confined. When lifted out of bed, and asked to endeavour to move either leg, he said it was quite useless, and did not even make the attempt. This is a point deserving of notice, because I believe it will be found that even when paraplegia is complete, and the mind unaffected, the patient will always make some attempt to move the limbs, though the effort be unavailing, or even productive of pain. In this case, the hesitation, or rather, I should say, the refusal to make the attempt, leads to the conclusion, that a want of inclination, rather than of voluntary power, lay at the bottom of the symptom. In saying this, I do not mean that the patient had *full* power if he had tried to move the limbs—the wasted condition of the muscles being in itself an impediment to their free action.

In asking him as to his history, he replied to all my questions in a very loud tone of voice, as if he were speaking to a deaf person; and on being assured that I could hear perfectly well, he said that he could not help speaking in that way, as he was suffering from nervous hysteria—the result of the use of large doses of opium, which he had taken for neuralgia, but had latterly left off, and that his crying out was quite uncontrollable on his part. But the loudness with which he spoke was not the only circumstance that attracted attention in this matter; it was evident that his voice was pitched in an artificial key also, the result of a direct and very laborious effort. He was anxious to make an impression upon me as a stranger, and, as happens not unfrequently in such cases, the very effort to sustain a part defeated itself. The rest of the time spent in the visit that day, was occupied by his endeavouring to prove that he should die if he were left in the hospital for want of the kind, assiduous attention of his own family. He said that he was accustomed to the greatest kindness, and that if he were left for a moment, or treated with neglect, he should be lost; and then appealing to my feelings, he added, he was sure, from the benevolence of my countenance, that I would not think of keeping him there, inasmuch as I must be convinced it was not a place adapted for his case. Informing him that I had not a single particle of compassion in my composition whenever duty was concerned, and that if he did not endeavour to control himself, so as not to annoy the other inmates of the house, effectual means would be taken to make him do so, I withdrew.

The next day I found that he had been roaring incessantly ever since my visit,—that he had not slept, and that he had taken no food, having resolved to starve himself to death. I confess I felt very much distressed at this account, and fully resolved in my own mind to insist, at whatever risk, on his being removed from the hospital. I determined, however, to conceal this intention on my part, and entering into his room, found him hoarse with shouting, and exhausted with his exertions and want of rest. The tone of his voice, however, was quite natural. After expostulating with him for some time, and informing him that, after the manner in which he had acted, it was impossible for him to return home, I ordered into the room a large tub filled with cold water, and pretended to be about to practise the cold effusion on his head, when I said I would give him one trial more, and that if he became noisy again, it should certainly be resorted to, I left him. Here I may remark, that his position in such an hospital as Sir P. Dun's, was anything but suitable for his proper treatment. The attendants, unskilled in managing such cases, spent the whole day and night in attempting to soothe him, and in prevailing on him to keep quiet, and to take food. But the more they coaxed him the worse he became. The mental condition, induced in early life, manifested itself strongly in this resistance to the wishes of matron and nurses; and the efforts they made, designed for his benefit only, created the greatest irritation, and exhausted his nervous energies. The proper course to be pursued in such cases, as I endeavoured to explain to them, but with too little effect, was to leave him very much to his own inclinations, to speak to him but little and while seeing that he wanted no necessary attention, to appear to take no notice of the great annoyance he was creating.

I lost no time in communicating to his relatives my conviction that it would be quite out of the question to keep him any longer in the hospital, both out of regard to his peculiar condition, and also in consequence of the intolerable annoyance he was occasioning to the inmates. But as they were particularly anxious, before taking any step for his removal, that I should see Dr. Eustace, who was acquainted with all the particulars of his case, and hear what he had to say about him, I called upon him that afternoon, and he assured me that "he was the biggest rogue in Ireland," that he knew he could control his outcries if he would, that he made every allowance for his shouting at a previous period, when he was suffering from pain, that his only object was to tire the attendants out, so as to induce them to send him home, to get rid of him, and that whenever he should find that method ineffectual, he would probably desist; and that certainly there was no fear of his starving himself to death. I said I fully concurred in all this, but that unless Mr. G. altered his habits very soon, I could not, in justice to the rest of the institution, retain him in the hospital, however much I might wish to accommodate his family.

On paying a second visit to the hospital that day, I learned that soon after my departure in the morning, finding, contrary to his expectations, that he had not succeeded in making an impression upon me sufficient to insure his removal, he called for food, and ate his meals heartily. I did not, on learning this circumstance, think it necessary or prudent to go into the room to see him.

The next morning I found him quite an altered man: he appeared cheerful, eat his food freely, and although he denied that he could at times help himself, so as not to cry out as he had done, seemed anxious to convince me that if he would only be let home, he would conduct himself in a rational and proper manner. I began to think that I had at least succeeded in effecting the object his friends had in view, and that he would soon be in a condition to return to his family without injury or inconvenience. But the expectation was delusive. On being informed that he must exhibit, by a longer probation, the power he possessed of controlling himself, before I could think of recommending his removal, he again became excited. He wanted me to promise that if he remained perfectly calm, and not troublesome for twenty-four hours, I would have him restored to his home; and when I said that I certainly would do so if he conducted himself properly for a week, he was not satisfied, and would not promise to make the attempt. It is obvious, that if he could have controlled himself for twenty-four hours, he ought certainly to have been equally able to do so for the longer period.

It is unnecessary to detail the daily changes that took place during his sojourn in the hospital. One or two circumstances, however, may be mentioned. One evening, when he was very noisy, and nurses and others had in vain attempted to quiet him, Mrs. Stephenson went in to him and succeeded in soothing him for a little; but in a quarter of an hour afterwards he sent for her a second time, and having asked him what he wanted, and ascertained that it was nothing particular, she said that she thought there would be no harm, as she saw he could not restrain himself, in his indulging his humour for crying out, and that it might perhaps do him good; that she would shut the doors, and leave him to himself, so that he would not disturb any one in the place. What was her amazement to hear him distinctly mutter, between his teeth, as he turned to his other side in bed, "Then I wont shout a word to please them."

After some days' residence in the hospital he fell into a desponding state as to his spiritual condition, complained that he had no one to read the Scriptures to him or pray for him, and that it was cruel to leave him any longer where he was, and where he could not have these advantages. I said that I would have some of his own ministers sent for, and that I was sure they would attend to all his wishes in that respect. It is needless to add that this did not satisfy him. He then said he knew he was lost to all eternity—that he had deserted his God, and that now, in just retribution, God had deserted him. He continued in this melancholy state for some days. Whether he really entertained this feeling or not I am unable to conjecture. It may have been a transient impression on his mind; it may have been merely another ruse to excite compassion in the minds of the spectators. It certainly left him before his removal from the hospital.

On the 11th of December he was very bad, roaring all night, and had become violent; he attempted to lacerate his person, tore a tooth out of his head, struck the nurse, injured the curtains and clothes of his bed, and in the morning, when somewhat subdued, said to me he was a degraded wretch, falling from one degree of degradation to a lower, and admitted the impropriety of his conduct, especially towards the nurse, whom he called his good, kind nurse. On my saying that now, in consequence of his conduct, he would require a probation of a month before I could recommend his return home, he said it would be of no use unless he could return *at once.* At a subsequent date he attempted to commit suicide by twisting some of the furniture of his bed tightly round his neck.

Finding, after repeated trials, that there was no prospect of his undergoing any improvement, I insisted on his friends having him removed from the hospital and on the 20th of December, 1852, he was transferred to the Friends' Retreat, at Donnybrook, as a clear case of moral insanity. On the day of his removal he promised that he would remain perfectly quiet for twenty-four hours in the hospital, provided I would promise that, at the end of that time, he should get a trial of six hours at his mother's house, and that if he then made the slightest noise, he would not object to his being taken to a lunatic asylum, or anywhere else that his friends might consider necessary.

When the application for this gentleman's admission to Sir P. Dun's was first made, it was stated, as a reason for selecting such a residence for him, that it was impossible for him to remain at home, that he required to be under constant and careful medical superintendence, and that he could not be received into any lunatic asylum, as there was not a physician in Ireland who would sign the necessary certificate, in consequence of the perfectly clear state of all his mental faculties. His disease was pronounced by the medical gentlemen in attendance to be a sort of chronic delirium tremens, brought on by the excessive use of opium at a time when he lived by himself, but latterly given up. Certainly to a casual observer there was no symptom of intellectual disorder. He laboured under no kind of delusion; he spoke rationally; his memory was clear and accurate; he had a perfect consciousness of the place he was in, of a person who attended him, and of every circumstance that happened to him. But are we justified, from these things, in concluding that there was no morbid condition of the understanding present all the time? That it was obscure I freely admit, but nevertheless I consider it was real. If I were asked wherein it consisted I would have no hesitation in affirming that it lay in a false conception of his own position in relation to his family, and the duties and responsibilities resulting therefrom. We have seen that for many years he had retired from the active engagements of business under the plea of illness, and had thrown himself for support upon the industry of his brother. It is quite true that he may have been really indisposed at the time, but, according to the statements I have heard from Dr. Eustace and his sisters, his illness was not of such a nature as to prevent his taking some part in the engagements of business, and at all events of evincing some interest in its success. Instead of that, he was known to say, "My family are well able to support me, and they must." Now I think it may be fairly asserted that any man who holds such a sentiment as this, takes a very false view of the obligations subsisting between the different members of a family, and is as justly to be considered in that respect insane as a person who fancies himself the Grand Turk, and claims the homage which is due to his imaginary rank. But if this be true of ordinary persons, it holds with peculiar force in the case of members of that religious denomination to which Mr. G. belonged, because I believe it to be a maxim in that respectable body that every individual should be trained up to habits of industry and usefulness, and should render himself useful in the world by the proper exercise of those talents with which he has been endowed. What renders this conduct in Mr. G. still more remarkable, is the fact that his family, shortly before his admission, had been reduced in circumstances by the failure of the firm with which they were connected, so that the whole duty of supporting the family was thrown upon the limited resources of his mother, whose income depended upon house rents.

There is another point, too, in which I think the reasoning faculties were clearly at fault. All Mr. G.'s endeavours, while he remained in the hospital, were, by one mode or another, to effect his return to his mother's residence. At one time it was by the excessive trouble he occasioned to the attendants, at another by the danger that appeared imminent to his own life, at a third by gentle persuasion or expostulation. Now, had he rightly exercised his judgment for the accomplishment of this object he could easily have succeeded; and I need hardly say that I, for one, would have used my utmost influence with his family to have persuaded them to comply with his earnest desire, had he made the attempt in the right way. All that he had to do, was to exercise the same self-control that he showed at times he was quite capable of for a longer period, and to manifest a desire to meet the very rational wishes of those in attendance upon him. But to this there was one very obvious objection in his mind, and I mention it because I conceive it illustrates the difficulty which persons who once place themselves in a false position must calculate on having subsequently to meet. It was this, Mr. G. had all along maintained that his screaming out was a matter of necessity, that his doing so was altogether involuntary, and that it was quite impossible for him to control himself. Had he at once met the wishes of those about him, he would have branded himself with the character of a deceiver, and of a troublesome wretch who wantonly gave annoyance to his kindest friends; and it may easily be conceived that to any one who has the least regard to a character for truth, it must be peculiarly humiliating to allow himself to be placed in such a position.

The whole question, however, hinges upon this point,—was it in Mr. G.'s power really to control himself when making this excessive noise or not? If it were, and he did not choose to do so, I think no person will hesitate to admit that his moral feelings must have been in a perverted condition, amounting to insanity; and on the other hand, if it were not, some reasonable explanation ought to be given of the intermissions that were observable in his noisiness. When a man is suffering acute pain, as in neuralgia, he will be forced to cry out when the paroxysm comes on, no matter who is in the room, or what he may be doing, and the moment of the seizure will be manifest by the suddenness and the severity of the patient's sufferings. In Mr. G.'s case nothing at all similar to this was observable. I got him to remain silent at my first interview, as well as subsequently, by addressing him steadily and in a tone of authority, and it was only when I ceased speaking, and allowed him time to reply, that he resumed his loud and unnatural mode of speaking. I have already alluded to the artificial key that his voice assumed on the first occasion. Such a circumstance never occurred subsequently. His voice, though often loud, was always pitched in its natural tone. Frequently he conversed in a quiet and gentle manner, but it was quite in vain that I attempted to make any impression upon him as to the unreasonableness of the conduct he was pursuing, and as to his perfect power of controlling his feelings if he were so disposed. At first he was not inclined to make any promise to restrain himself, declaring his utter inability to do so, but subsequently he was quite ready enough to accede to the proposal, providing a corresponding promise were made to him, to comply with his wishes on his keeping to his engagement.

In conclusion, I would sum up the observations I have to make on this case, by saying that I look upon it as a case of moral insanity, and that I found this opinion on the following grounds:—

1. There was a total disregard to the feelings of others in his constant and loud outcries, which he manifested no desire to restrain or put a stop to.

2. This was further displayed in his unceasing demand for personal attentions at all hours of the day or night.

3. In the little concern that he exhibited in being thrown as a burden upon the other members of his family when they were themselves involved in pecuniary difficulties.

4. In the injuries he inflicted on himself, and in his attempt to commit suicide by strangulation.

5. In his never attempting to conciliate the opposition of his friends to his return home, by expressions of regret for his past annoyance, and attempts at subsequent amendment.

